# Erythrocyte DHA/EPA Ratio Surpasses Its Individual Fatty Acid Levels in Predicting Metabolic Syndrome in Chinese Adults: A Prospective Study

**DOI:** 10.3390/nu17061096

**Published:** 2025-03-20

**Authors:** Pinning Feng, Yan Yan, Hanzu Chen, Dongmei Ru, Xinyue Wang, Yuming Chen

**Affiliations:** 1 Department of Epidemiology, School of Public Health, Sun Yat-Sen University, Guangzhou 510080, China; fengpn@mail.sysu.edu.cn (P.F.); yany36@mail2.sysu.edu.cn (Y.Y.); chenhz9@mail2.sysu.edu.cn (H.C.); rudm@mail2.sysu.edu.cn (D.R.); wangxinyue230@163.com (X.W.); 2 Department of Clinical Laboratory, The First Affiliated Hospital of Sun Yat-Sen University, Guangzhou 510062, China

**Keywords:** eicosapentaenoic acid, docosahexaenoic acid, erythrocyte, metabolic syndrome, prospective study

## Abstract

**Background:** The individual roles of docosahexaenoic acid (DHA) and eicosapentaenoic acid (EPA) in mitigating metabolic syndrome (MetS) have been well-documented. However, the significance of their ratio (DHA/EPA) in erythrocytes concerning MetS remains underexplored. This study evaluated the association between the DHA/EPA ratio and MetS including its separate components. **Methods:** This community-based prospective cohort study involved 3497 participants aged 40 to 75 years enrolled in the Guangzhou Nutrition and Health Study (GNHS) from 2008 to 2013 in China. Baseline erythrocyte fatty acids were quantified using gas chromatography. Over a 12-year follow-up, MetS status was reassessed triennially, identifying 766 new MetS cases among the 2111 participants initially free of the syndrome. The study examined both cross-sectional and prospective relationships of EPA, DHA, and the DHA/EPA ratio with both the presence and incidence of MetS alongside its individual components. **Results:** Multivariable cross-sectional analyses revealed that the odds ratios (ORs) and 95% confidence intervals (CIs) for MetS prevalence in quartile 4 (vs. 1) were 1.32 (1.07, 1.62) for EPA, 0.52 (0.40, 0.66) for DHA, and 0.66 (0.52, 0.83) for the DHA/EPA ratio (all *P*-trend < 0.05). Analogous patterns were observed for associations with hyperlipidemia, hypertension, and hyperglycemia. In the prospective analysis, higher DHA/EPA ratios and lower EPA levels were associated with reduced 12-year incidence of MetS and hyperglycemia. Hazard ratios (HRs) and 95% CIs for MetS, comparing the extreme quartiles, were 1.26 (1.02, 1.55) for EPA and 0.75 (0.60, 0.94) for the DHA/EPA ratio. Additionally, DHA was inversely associated with the incidence of hypertension. **Conclusions:** The study highlights a consistent inverse relationship between the DHA/EPA ratio and both the prevalence and risk of MetS. These findings underscore the potential utility of the DHA/EPA ratio as a predictive marker or therapeutic target in MetS management.

## 1. Introduction

Metabolic syndrome (MetS) presents as a cluster of metabolic disorders, including hypertension, hyperglycemia, dyslipidemia, and abdominal obesity [1], which significantly increases the risk of developing type 2 diabetes and cardiometabolic diseases [2]. Given its prevalence, which affects 20–30% of the global population and up to 33.9% in China, MetS has become a significant public health challenge [3,4]. Early detection and intervention play a vital role in reducing cardiovascular disease risk associated with MetS [5]. The effective management of MetS focuses on modifiable risk factors, with lifestyle and dietary changes at the forefront. Omega-3 long-chain polyunsaturated fatty acids (PUFAs), notably eicosapentaenoic acid (EPA) and docosahexaenoic acid (DHA), are celebrated for their extensive health benefits including their potential to mitigate MetS risk [6,7]. These fatty acids are recognized for their antioxidant properties, enhancing enzyme activity to alleviate oxidative stress and improving MetS outcomes [8,9]. Moreover, EPA and DHA are precursors to resolvins, anti-inflammatory mediators instrumental in ceasing inflammatory processes [10,11,12]. Research has consistently shown the inverse relationship between PUFAs, including EPA and DHA, and MetS prevalence, especially within Chinese adult populations [13]. Furthermore, clinical trials have confirmed the efficacy of omega-3 FA supplements, particularly those rich in EPA and DHA, in reducing blood pressure and heart rates compared with placebos [14]. However, it remains unclear whether EPA and DHA have interchangeable effects on MetS or whether a specific ratio of them is more appropriate for metabolic health.

The DHA/EPA ratio has gained increasing attention as studies suggest that adjustments to this ratio may significantly affect the cardiovascular risk factors and inflammation [15,16]. For individuals with type 2 diabetes, an EPA/DHA ratio exceeding 1.5 has been associated with greater triglyceride (TG) reductions [16]. Animal studies have revealed that specific EPA to DHA ratios (1:1 and 2:1) effectively reduce plasma fatty acids, triglycerides, cholesterol, LDL, and C-reactive protein levels [17]. Notably, a mixture of EPA and DHA has shown superior benefits in reducing the fasting blood glucose, plasma insulin concentrations, and HOMA-IR compared with administering either fatty acid alone [18]. Studies using Wistar and SHROB rats indicated that EPA to DHA ratios of 1:1 and 2:1 were particularly effective in eliciting anti-inflammatory responses [19,20]. Despite these findings, currently, no consensus on the most beneficial EPA to DHA ratio for combating MetS and its components has been reached.

This research aimed to address this gap by investigating the association between circulating EPA, DHA, and the DHA/EPA ratio on the prevalence and incidence of MetS and its components in a Chinese cohort.

## 2. Materials and Methods

### 2.1. Study Participants

This investigation was conducted as part of the Guangzhou Nutrition and Health Study (GNHS, www.clinicaltrials.gov [No. NCT03179657]), a community-based prospective cohort study conducted in urban Guangzhou, China. The GNHS enrolled 4048 participants aged 40–75 years from 2008 to 2013, with 3497 (2390 women and 1107 men) participants having erythrocyte membrane fatty acids measured at baseline. Participants were subsequently followed up triennially over a 12-year follow-up.

**Figure 1:**
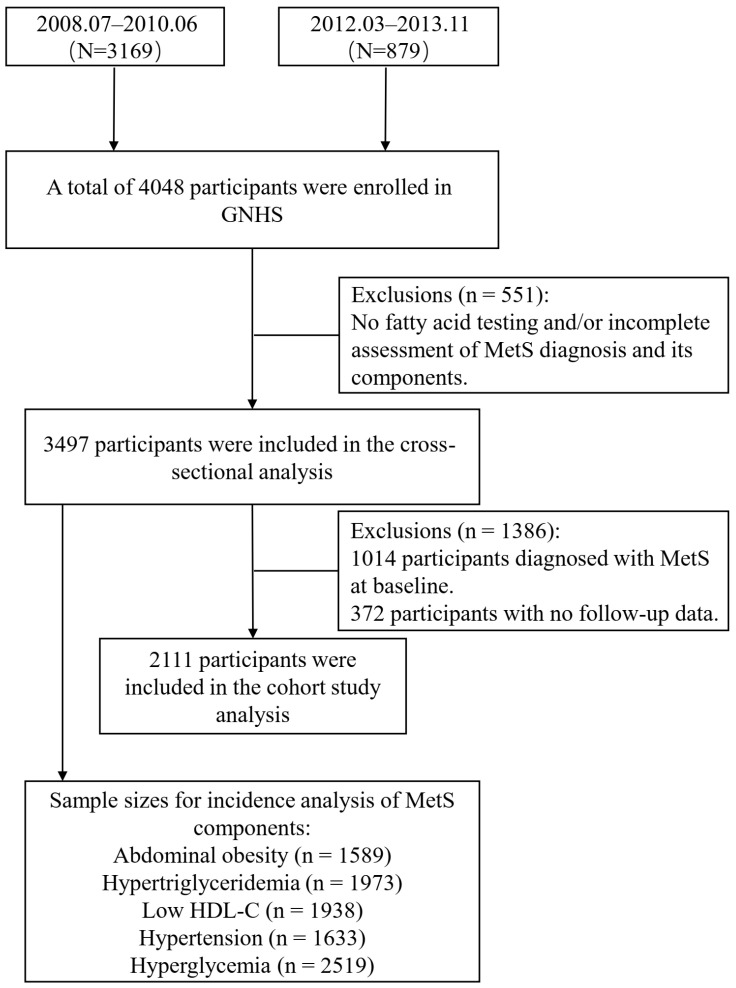
Inclusion flowchart for the study population.

MetS status was evaluated during each follow-up visit. Among the 2111 participants who were initially free of MetS, 766 new cases were identified during the follow-up period. A total of 3497 and 2111 participants were enrolled in the cross-sectional and prospective analyses, respectively. The study protocol was approved by the Ethics Committee of the School of Public Health at Sun Yat-Sen University. Prior to participation, comprehensive written informed consent was obtained from all individuals involved. The assessment process encompassed biospecimen collection, detailed physical examinations, and exhaustive questionnaires to gather extensive data. The specific process is illustrated in Figure 1.

### 2.2. Data Collection

Data were collected through structured questionnaires during face-to-face interviews conducted by trained medical personnel. These questionnaires meticulously gathered the baseline sociodemographic data, lifestyle factors (including smoking status, alcohol consumption, physical activity levels, and dietary habits), current health status, and medical history. Smoking status was classified into current smokers and non-smokers, while current drinkers were defined as those who consumed alcohol at least once per week for more than six months. Physical activity was evaluated through a comprehensive 19-item questionnaire, which quantified the metabolic equivalent (MET) hours per day [21].

Dietary intake was evaluated using a validated 79-item food frequency questionnaire (FFQ) that inquired about the frequency of food and beverage consumption over the preceding year, categorized as “never”, “per month”, “per week”, or “per day”, along with the average portion sizes. Daily nutrient intake was then calculated according to the Chinese Food Composition Table 2002 [22].

Anthropometric measurements, including weight, height, waist, and hip circumference, were conducted by trained staff. Body mass index (BMI) was calculated using the formula: weight (kg) divided by height (m^2^). Waist circumference measurements were taken at the midpoint between the lower rib margin and the iliac crest, while the hip circumference was measured at the widest part over the buttocks. Blood pressure (BP) readings were taken twice on the right arm using a standard mercury sphygmomanometer after a minimum of 5-min rest. A third measurement was conducted if the initial two BP readings varied by more than 4 mmHg for systolic BP (SBP) or 3 mmHg for diastolic BP (DBP). The average of the two (or three, if necessary) measurements was utilized for data analysis.

### 2.3. Laboratory Measurements

Venous blood samples were collected from participants after an overnight fast at the baseline visit for comprehensive lipidomic profiling. Serum levels of the total cholesterol (TC), triglycerides (TG), high-density lipoprotein cholesterol (HDL-c), low-density lipoprotein cholesterol (LDL-c), and fasting blood glucose were measured using a Hitachi 7600–010 automated analyzer (Hitachi, Tokyo, Japan). The intra-assay coefficients of variation for these measurements were 2.52% for fasting glucose, 4.67% for LDL-c, 3.47% for HDL-c, 2.17% for TC, and 2.86% for TG, indicating high reliability and precision.

Fatty acid compositions of erythrocyte membranes were determined via gas chromatography analysis. Fatty acids were initially extracted from erythrocytes using a chloroform/methanol solution, followed by extracting the resultant fatty acid methyl esters with hexane, which contained 0.05% butylated hydroxytoluene. These hexane extracts were then sealed under nitrogen before gas chromatography, conducted using an Agilent 7890a gas chromatograph (Agilent Technologies Inc., Palo Alto, CA, USA) equipped with a 60-m Agilent DB-23 column (Agilent Technologies Inc., Palo Alto, CA, USA). Fatty acids were identified by comparing the retention times with purified fatty acid standards (Nu-ChekPrep Inc., Waterville, MN, USA) and quantified as area percentages. The coefficients of variation for EPA and DHA measurements were 14.6% and 11.4%, respectively.

### 2.4. Definition of Metabolic Syndrome

MetS was defined following the criteria set forth by the modified International Diabetes Federation, the American Heart Association, and the National Heart, Lung, and Blood Institute [23]. The criteria included: (1) abdominal obesity, defined by waist circumference of ≥90 cm for men or ≥80 cm for women; (2) elevated blood pressure, defined as SBP ≥ 130 mmHg and/or DBP ≥ 85 mmHg, or the use of antihypertensive medication in participants with a history of hypertension; (3) hyperglycemia, characterized by fasting glucose levels ≥ 5.6 mmol/L or the use of anti-diabetic agents or insulin; (4) reduced HDL-c, with levels < 1.0 mmol/L in men and <1.3 mmol/L in women; and (5) elevated plasma triglycerides, ≥1.7 mmol/L. An individual was classified as having MetS if at least three of these conditions were present.

### 2.5. Data Analysis

We reported the summary statistics for the demographic and clinical variables of the participants, presenting continuous variables as median (interquartile range) and categorical variables as percentages (%). To assess the differences between groups for continuous variables, we employed either the Wilcoxon rank sum test or Student’s *t*-test, depending on the data distribution. For categorical variables, the chi-square test was used for comparison.

Participants were divided into quartiles based on their erythrocyte levels of EPA, DHA, and the DHA/EPA ratio, stratified by sex. In the cross-sectional analysis of the baseline data, multivariable logistic regression models were then utilized to calculate the odds ratios (ORs) and 95% confidence intervals (CIs) for the prevalence of MetS and its individual components across the 2nd, 3rd, and 4th quartiles of EPA, DHA, and the DHA/EPA ratio levels, using the lowest quartile as the reference group. For the 12-year longitudinal analysis of the incidence of MetS and its components, Cox proportional hazards regression models were employed to derive both the unadjusted and adjusted hazard ratios (HRs) and 95% CIs. Basic demographic characteristics (i.e., gender and age) were included as covariates in Model 1. Model 2 additionally adjusted for three categories of potential confounders: socioeconomic factors (education, marital status, income), lifestyle factors (tea, smoking, alcohol, multivitamins, physical activity), and dietary factors (energy intake, fiber, saturated fatty acids [SFAs]).

Statistical significance was determined based on two-sided *p*-values of 0.05 or less. Data analysis was performed utilizing R (version 4.1.3, R Core Team).

## 3. Results

### 3.1. Characteristics of the Participants

The study included 3497 individuals, comprising 1107 men and 2390 women, with an average age of 58 years. Comparative analysis of the baseline characteristics between participants with and without MetS revealed significant differences in the lipid profiles. Specifically, individuals with MetS exhibited significantly higher erythrocyte TG and TC levels and lower HDL-c levels than their those without MetS (all *p* < 0.05). Furthermore, the erythrocyte percentage of EPA was significantly higher in the MetS group. In contrast, the DHA levels and DHA/EPA ratio were lower compared with the non-MetS group (all *p*< 0.001) (Table 1).

### 3.2. Associations of EPA, DHA, and the DHA/EPA Ratio with the Prevalence of MetS and Its Components

**Figure 2:**
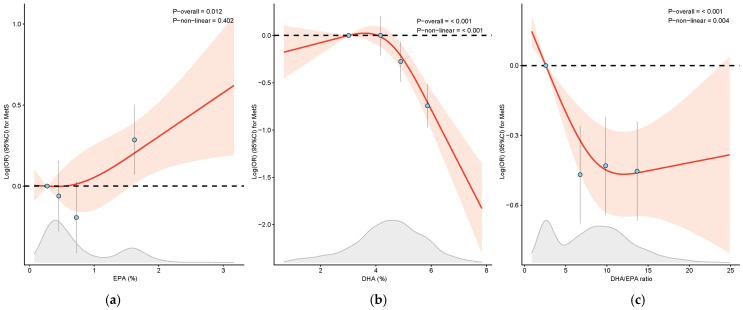
Restrictive cubic spline analysis of EPA (**a**), DHA (**b**), and the DHA/EPA ratio (**c**) with MetS prevalence. The red lines and blue dots represent the log-transformed odds ratio (OR). Meanwhile, the light red area and the error bars represent the 95% confidence intervals (CIs). The grey area illustrates the distribution of the study population by specific fatty acids. Abbreviation: EPA, eicosapentaenoic acid; DHA, docosahexaenoic acid.

In our cross-sectional analysis, we discovered a significant positive association between the EPA levels and MetS prevalence, whereas the DHA levels and DHA/EPA ratio were inversely associated with MetS across all models (Table 2). The multivariate-adjusted ORs for MetS prevalence were as follows: 1.32 (95% CI 1.07, 1.62) for EPA, 0.48 (95% CI 0.39, 0.60) for DHA, and 0.64 (95% CI 0.52, 0.79) for the DHA/EPA ratio when comparing the highest quartile to the lowest in Model 2. As the DHA/EPA ratio increased from a median of 2.60 in Q1 to 6.76, 9.86, and 13.61 in quartiles Q2–Q4, respectively, the ORs demonstrated a decreasing trend to 0.66, 0.67, and 0.64 (all *P*-trend < 0.001). Restricted cubic spline analysis revealed a linear dose–response association between the EPA levels and the odds of MetS (*P_-_*_overall_ = 0.012, *P-*_nonlinear_ = 0.402), but a nonlinear correlation between the DHA and DHA/EPA ratio and MetS odds (*P_-_*_overall_ < 0.001, *P_-_*_nonlinear_ < 0.05), as illustrated in Figure 2.

When examining the individual components of MetS, elevated EPA levels were significantly associated with an increased prevalence of hypertension and hyperglycemia (*P*-trend ≤ 0.01). Conversely, both the DHA levels and DHA/EPA ratio were inversely correlated with the prevalence of hypertriglyceridemia (HTG), hypertension, and hyperglycemia (all *P*-trend < 0.005) (Table 3). An optimal DHA/EPA ratio (>6.76), observed in quartiles Q2–Q4, was associated with lower ORs for HTG, hypertension, and hyperglycemia, suggesting the potential protective effects of a higher DHA/EPA ratio against MetS and its components.

### 3.3. Associations of EPA, DHA, and the DHA/EPA Ratio with 12-Year Incidence of MetS and Its Components

**Figure 3:**
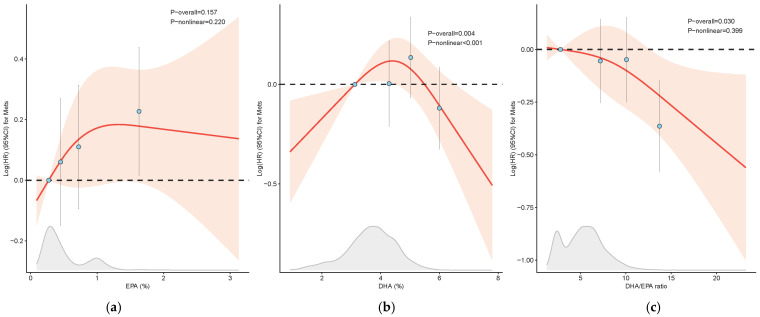
Restrictive cubic spline analysis of EPA (**a**), DHA (**b**), and the DHA/EPA ratio (**c**) with MetS incidence. The red lines and blue dots represent the log-transformed hazards ratio (HR). Meanwhile, the light red area and the error bars represent the 95% confidence intervals (CIs). The grey area shows the distribution of participants by specific fatty acids. Abbreviations: EPA, eicosapentaenoic acid; DHA, docosahexaenoic acid.

The 12-year longitudinal analysis demonstrated that an increased incidence of MetS was associated with higher levels of EPA and lower DHA/EPA ratios, with *P*-trends of 0.032 and 0.002, respectively, compared with DHA (Table 4). Specifically, the HRs for the highest quartile versus the lowest quartile, adjusted for confounders in Model 2, were 1.26 (95% CI: 1.02, 1.55) for EPA and 0.70 (95% CI: 0.56, 0.86) for the DHA/EPA ratio. Additionally, restrictive cubic spline analysis revealed a linear correlation between the DHA/EPA ratio and the risk of MetS (*P_-_*_overall_ = 0.030, *P_-_*_nonlinear_ = 0.399), while a nonlinear correlation was observed between the DHA levels and MetS risk (*P_-_*_overall_ = 0.004, *P_-_*_nonlinear_ < 0.001), as shown in Figure 3.

Further analysis of the components of MetS revealed that higher EPA levels and lower DHA/EPA ratios were associated with and increased risk of hyperglycemia. Higher DHA levels were inversely associated with hypertension risk (all *P*-trend < 0.05). The HRs for hyperglycemia in the highest versus the lowest quartile were 1.24 (95% CI: 1.02, 1.50) for EPA and 0.79 (95% CI: 0.65, 0.96) for the DHA/EPA ratio. The corresponding HR for hypertension was 0.76 (95% CI: 0.61, 0.96) for DHA (Table 5).

## 4. Discussion

Our prospective cohort study provides compelling evidence regarding the relationship between fatty acid profiles, particularly the DHA/EPA ratio and MetS. Both cross-sectional and longitudinal analyses consistently demonstrated a beneficial association between the DHA/EPA ratio and both the presence and incidence of MetS. Interestingly, the beneficial association with DHA was confirmed in the cross-sectional analyses, but was not observed in the prospective analyses. Moreover, in contrast to some of the existing literature, our study uniquely identified a positive correlation between the erythrocyte levels of EPA and both the presence and incidence of MetS. These observed differential effects suggest that a higher DHA/EPA ratio may serve as a more effective biomarker for the potential intervention and prediction of MetS than individual levels of DHA or EPA.

### 4.1. EPA and Metabolic Health

Despite the recently documented beneficial effects of a high dose of total *n*-3 fatty acids on MetS, evidence concerning the relationship between individual *n*-3 fatty acids and MetS is limited and inconsistent. In a cross-sectional study of 3072 Chinese individuals, a significant negative correlation was found between EPA, DHA, and MetS [13], a finding not consistent with our study. However, two other studies encompassing Asian and American populations revealed that EPA appears to be unrelated to MetS [24,25]. Several factors, such as the sample size, study design, follow-up period, and the control for potential confounding variables in the adjusted models, may explain the discrepancies in these findings. Further research is warranted to validate our findings, and the underlying mechanism requires additional evaluation in future studies.

Some studies have explored the relationship between EPA and components of MetS. Research indicates that EPA does not affect patients with hypertensive diabetes. In healthy individuals, EPA can improve blood pressure, but its effectiveness is not as strong as that of DHA [26]. Another similar study in young healthy men and women in Australia revealed comparable results. The EPA treatment group showed no effect on dynamic blood pressure or heart rate in men with hyperlipidemia, whereas the DHA group was able to reduce both dynamic blood pressure and heart rate [27]. In our study, we found that higher concentrations of EPA were associated with an increased risk of hyperglycemia. However, previous studies have suggested that EPA may have beneficial effects in improving insulin sensitivity. Specifically, EPA has been shown to mitigate insulin resistance by reducing adipose tissue inflammation [28,29,30]. Thus, higher EPA concentrations are not directly linked to an increased risk of hyperglycemia; on the contrary, they may contribute to improved metabolic health under certain conditions. The observed association between elevated EPA levels and hyperglycemia risk may be explained by several factors. First, an imbalance in the EPA-to-DHA ratio could play a role, as high EPA concentrations may correspond to relatively low DHA levels, potentially leading to unfavorable metabolic effects [31]. Second, interindividual variability, such as genetic background, dietary habits, and other individual factors, may influence the metabolic effects of EPA [32,33,34].

### 4.2. DHA and Metabolic Health

A Chinese cohort study showed a negative correlation between erythrocyte DHA and MetS [24], and a meta-analysis further suggested that high levels of DHA in the diet or blood may have a positive impact on MetS risk [25]. In addition, a cross-sectional study conducted in China reported a negative correlation between DHA and MetS [13]. These findings are largely consistent with our results, as noted above.

Evidence indicates that DHA is more effective than EPA in reducing blood pressure and heart rate [26,27] and inhibiting vasoconstriction [35]. These results align closely with the findings of our study: higher DHA, but not EPA, significantly reduced the risk of hypertension. DHA can lower blood pressure to a greater extent, possibly by altering the composition of membrane fatty acids and potentially accelerating the release of ATP from the endothelial cells, which is linked to a decrease in plasma norepinephrine [36]. Furthermore, DHA can act as a lipid regulator to prevent thromboxane-induced contractions and may restore the balance of vasoconstrictors/vasodilators after the disruption of normal nitric oxide-related processes [37].

Consistent with our findings, previous studies have demonstrated a negative association between DHA and the risk of hyperglycemia [24]. A recent review indicated that DHA likely improves glucose use by the brain. By regulating the expression of glucose transporter 1 (GLUT1), glucose uptake and utilization are promoted [38]. In support of our findings, previous studies have shown a negative association between DHA and hyperglycemia risk [24]. In addition, DHA supplementation significantly elevated insulin levels in overweight men with hyperlipidemia [39]. These results may suggest that the differing effects of DHA and EPA observed across studies may be limited to variations in the concentration of these fatty acids. The distinct roles of DHA and EPA in regulating glucose metabolism warrant further investigation to better understand their relationship with the risk of MetS.

### 4.3. DHA to EPA Ratio and Metabolic Health

While previous studies have mainly focused on DHA or EPA individually, emerging evidence underscores the importance of their combined effects and specific ratios in managing MetS. This approach is supported by several key findings. EPA (20 carbons) and DHA (22 carbons) have different molecular structures, leading to distinct biological roles. DHA concentrates in lipid rafts [40] modulate the membrane physical properties and signaling pathways, while EPA influences the membrane fluidity and cholesterol organization [41]. Recent evidence suggests that EPA significantly enhances the cellular antioxidant capacity, potentially by improving mitochondrial function and promoting biosynthesis [42]. These mechanisms suggest that EPA may have more direct cellular effects in combating oxidative stress. This differential impact on oxidative stress regulation may contribute to the distinct physiological effects observed with varying EPA/DHA ratios.

Both EPA and DHA reduce inflammation through complementary mechanisms: EPA primarily reduces the secretion of tumor necrosis factor-α (TNF-α), while DHA inhibits interferon-γ (IFN-γ) release [43]. These synergistic anti-inflammatory effects may collectively influence lipid metabolism, explaining the metabolic benefits observed with combined supplementation. 

A few animal studies have examined the effects of different DHA/EPA ratios on cardiometabolic markers and generated inconsistent results. In an experiment involving apoE knockout (apoE^−^/^−^) mice, interventions with different DHA/EPA ratios (2:1, 1:1, and 1:2) showed that the 2:1 group exhibited lower levels of TC, LDL-C, tumor necrosis factor-alpha (TNF-α), and aortic reactive oxygen species (ROS) [44]. Conversely, the same research team, using a C57BL/6J mouse model, found that the group with the lowest DHA/EPA ratio (1:2) had reduced TG, TC, and LDL-C levels, but higher hepatic TC and TG levels [45]. Two studies showed that benefits were observed in a moderated ratio of DHA/EPA. One study indicated that a diet of 1:1 DHA/EPA (compared with 2:1 or 1:2) improved the oxidative stress parameters, plasma antioxidant capacity, and cardiovascular risk factors in rats [19]. Another study demonstrated that the 1.5:1 ratio of DHA/EPA, ranging from 3:1 to 1:3, indicated that it was the most effective in preventing insulin resistance in C57BL/6J mice [30].

Few studies have directly compared the effects of different EPA/DHA ratios within the same population or investigated the impact of interventions with varying EPA/DHA ratios on cardiovascular health outcomes. Although studies have shown that supplementation with 4 g/day of EPA alone significantly reduces cardiovascular disease (CVD) events (HR: 0.75, 95% CI 0.68–0.83), other randomized controlled trials (RCTs) using combined EPA and DHA supplementation (doses ranging from ≤1 g/day to 4 g/day) have not demonstrated statistically significant clinical benefits [46]. A meta-regression analysis of RCTs examined the impact of varying EPA/DHA ratios across different trials on cardiometabolic markers and found that higher EPA/DHA ratios were associated with increased systolic blood pressure (SBP) and reduced C-reactive protein (CRP) levels, but no significant associations were observed with triglycerides (TGs), total cholesterol (TC), high-density lipoprotein cholesterol (HDL-C), or low-density lipoprotein cholesterol (LDL-C) [16]. Through animal models, it has been shown that the anti-hypertensive effects of EPA and DHA may be related to improving endothelial vasodilation function [47,48], reducing the pressor responsiveness of resistance vessels [48,49], and increasing vascular compliance [50].

Overall, research on the relationship between the DHA/EPA ratios and health outcomes remains limited, with inconsistent findings. Further studies are needed to address these scientific questions and provide clearer insights.

## 5. Strengths and Limitations

Our 12-year prospective study design represents a significant strength, providing a robust framework for evaluating the impact of the DHA/EPA ratio on the risk of MetS and its components. This extended observation period enhanced the reliability of our findings, capturing the long-term effects of fatty acid profiles on metabolic health. Furthermore, our comprehensive analysis delved into the DHA/EPA ratio’s influence not only on the presence of MetS, but also on its incidence over time, offering valuable insights into its potential role in managing and preventing metabolic health deterioration. The study’s large cohort size further strengthened the validity of our observed associations, ensuring that our conclusions reflect broader population trends.

However, our study was not without limitations. A key limitation was the single measurement of the erythrocyte membrane EPA and DHA levels at the baseline, which may not capture the temporal variations in the fatty acid profiles resulting from dietary or lifestyle changes over the 12-year follow-up period. This could lead to exposure misclassification and potentially weaken the observed associations. While the core conclusions of this study are supported by the laboratory-measured parameters, we acknowledge the inherent limitations of questionnaire-based dietary assessments, which include the potential for dietary intake misclassifications, potential recall bias in self-reported food intake, and the imperfect categorization of distinct food sources into groups. Moreover, the focus on a Chinese population may limit the generalizability of our findings across different ethnic or racial groups, given the possible variations in genetic, dietary, and lifestyle factors. Despite adjusting for a wide range of confounders, the potential for residual confounding from unmeasured or unknown factors remains. To address these limitations and validate our findings, future research, particularly randomized controlled trials involving diverse demographic and ethnic groups, is essential to further clarify the causal relationships between the DHA/EPA ratio and MetS and verify the applicability of our results.

## 6. Conclusions

Our study illuminates a notable and consistently beneficial association of the DHA to EPA ratio with both the presence and incidence of MetS, a pattern not observed with the individual levels of EPA and DHA in this population. Intriguingly, higher levels of EPA were found to correlate with an increased risk for both the prevalence and incidence of MetS. These differential impacts emphasized in our findings underscore the potential of the DHA/EPA ratio as a superior biomarker for both the management and prediction of MetS, outperforming the individual fatty acid levels.

## Figures and Tables

**Table 1:** Baseline characteristics of the total study participants.

Characteristics	Total Participants (n = 3497)	Non-MetS (n = 2483)	MetS (n = 1014)	*P*
Age, year	58.00 (54.00, 63.00)	57.00 (54.00, 62.00)	60.00 (55.00, 65.30)	<0.001
gender n (%)				<0.001
women	2390 (68.34)	1651 (66.49)	739 (72.87)	
men	1107 (31.65)	832 (33.51)	275 (27.13)	
Current smoker n (%)	631 (18.04)	417 (16.79)	160 (15.78)	0.494
Current drinker n (%)	239 (6.83)	164 (6.60)	75 (7.39)	0.443
Education level n (%)				<0.001
Middle school	1056 (30.19)	703 (28.31)	353 (34.81)	
High school or professional college	1591 (45.49)	1171 (47.16)	420 (41.42)	
University	850 (24.30)	609 (24.53)	241 (23.77)	
Household income (Chinese Yuan/month/person) n (%)				0.012
<1500	1729 (49.44)	1254 (50.50)	475 (46.84)	
1500–3000	851(24.34)	613 (24.69)	238 (23.47)	
≥3000	917 (26.22)	616 (24.81)	301 (29.68)	
Physical activity MET/d	35.60 (30.50, 49.70)	36.20 (30.90, 52.20)	34.20 (29.80, 45.20)	<0.001
BMI kg/m^2^	23.20 (21.20, 25.30)	22.40 (20.60, 24.20)	25.20 (23.40, 26.90)	<0.001
Waist circumference cm	83.00 (77.00, 89.30)	80.30 (75.00, 86.50)	89.00 (84.00, 94.00)	<0.001
SBP mmHg	122 (110, 135)	120 (110, 130)	131 (121, 141)	<0.001
DBP mmHg	79 (70, 83)	76 (70, 80)	80 (75, 89)	<0.001
Fasting glucose mmol/L	4.70 (4.30, 5.20)	4.60 (4.20, 5.00)	5.10 (4.50, 5.82)	<0.001
Serum lipids mmol/L				
TG mmol/L	1.31 (0.93, 1.83)	1.12 (0.84, 1.49)	1.97 (1.44, 2.72)	<0.001
TC mmol/L	5.44 (4.75, 6.16)	5.41 (4.75, 6.11)	5.49 (4.77, 6.26)	0.044
HDL-c mmol/L	1.36 (1.16, 1.59)	1.45 (1.25, 1.67)	1.16 (1.01, 1.29)	<0.001
LDL-c mmol/L	3.54 (2.98, 4.13)	3.54 (3.02, 4.11)	3.55 (2.90, 4.19)	0.579
Erythrocyte FA composition% of total fatty acids				
EPA(C20:5)%	0.56 (0.37, 1.26)	0.55 (0.36, 1.07)	0.60 (0.38, 1.46)	<0.001
DHA(C22:6)%	4.54 (3.73, 5.31)	4.63 (3.80, 5.43)	4.33 (3.65, 5.02)	<0.001
DHA/EPA ratio%	8.38 (4.03, 11.36)	8.64 (5.03, 11.50)	7.75 (2.97, 10.87)	<0.001
Dietary daily intakes				
DHA(C22:6) mg/d	0.03 (0.02, 0.05)	0.03 (0.02, 0.05)	0.03 (0.02, 0.05)	0.132
EPA(C20:5) mg/d	0.02 (0.01, 0.03)	0.02 (0.01, 0.03)	0.02 (0.01, 0.03)	0.039
DHA/EPA intake ratio%	1.60 (1.33, 2.00)	1.57 (1.33, 2.00)	1.67 (1.33, 2.00)	0.148
Energy intake kcal/d	1687 (1408, 2068)	1708 (1432, 2098)	1645 (1352, 1988)	<0.001
Fiber g/d	10.54 (8.08, 13.64)	10.61 (8.15, 13.74)	10.22 (7.95, 13.19)	0.018
SFA g/d	14.10 (10.50, 18.70)	14.20 (10.70, 19.00)	13.70 (10.10, 18.00)	0.002

Data are presented as median (P25, P75) or n (%). n represents the number of individuals that fall into that variable category. Abbreviations: MetS, metabolic syndrome; BMI, body mass index; SBP, systolic blood pressure; DBP, diastolic blood pressure; TG, triacylglycerol; TC, total cholesterol; HDL-c, HDL cholesterol; LDL-c, LDL cholesterol; EPA(C20:5), eicosapentaenoic acid (20:5); DHA(C22:6), docosahexaenoic acid(C22:6); SFAs, saturated FAs.

**Table 2:** Multivariable-adjusted ORs (95% CIs) for the presence of metabolic syndrome by quartiles of erythrocyte polyunsaturated fatty acids.

Variables	ORs (95% CI) by Quartiles of *n*-3 PUFA Concentrations				*P*-Trend
	Quartile 1	Quartile 2	Quartile 3	Quartile 4	
EPA(C20:5)					
Median (%)	0.27	0.45	0.72	1.62	
Case/n	240/875	239/874	218/873	317/875	
Crude Model	1.00	1.00 (0.81, 1.23)	0.88 (0.71, 1.09)	1.50 (1.23, 1.84)	<0.001
Model 1	1.00	0.99 (0.80, 1.23)	0.86 (0.70, 1.07)	1.36 (1.10, 1.67)	0.016
Model 2	1.00	0.98 (0.79, 1.22)	0.86 (0.69, 1.07)	1.32 (1.07, 1.62)	0.033
DHA(C22:6)					
Median	3.02	4.16	4.89	5.86	
Case/n	292/875	307/874	243/873	172/875	
Crude Model	1.00	1.08 (0.89, 1.32)	0.77 (0.63, 0.94)	0.49 (0.39, 0.61)	<0.001
Model 1	1.00	1.05 (0.86, 1.29)	0.71 (0.58, 0.88)	0.48 (0.39, 0.60)	<0.001
Model 2	1.00	1.04 (0.85, 1.28)	0.70 (0.57, 0.86)	0.48 (0.39, 0.60)	<0.001
DHA/EPA ratio					
Median	2.60	6.76	9.86	13.61	
Case/n	326/875	236/874	229/873	223/875	
Crude Model	1.00	0.62 (0.51, 0.76)	0.60 (0.49, 0.73)	0.58 (0.47, 0.71)	<0.001
Model 1	1.00	0.65 (0.53, 0.80)	0.65 (0.53, 0.80)	0.63 (0.51, 0.77)	<0.001
Model 2	1.00	0.66 (0.53, 0.81)	0.67 (0.54, 0.83)	0.64 (0.52, 0.79)	<0.001

Abbreviations: PUFAs, polyunsaturated fatty acids; EPA(C20:5), eicosapentaenoic acid (20:5); DHA(C22:6), docosahexaenoic acid(C22:6). Model 1: adjusted for age, gender; Model 2: further adjusted for education, marital status, income, tea drinking, smoking status, alcohol drinking, multivitamin use, physical activity, daily energy intake, dietary fiber, and SFAs.

**Table 3:** Multivariable-adjusted ORs (95% CIs) for the prevalence of metabolic syndrome components by quartiles of erythrocyte polyunsaturated fatty acids.

Variables	Abdominal Obesity	HTG	LOW HDL-C	Hypertension	Hyperglycemia
Cases/n	1652/3497	1168/3497	1193/3497	1628/3497	549/3497
EPA(C20:5)					
Quartile 1	1.00	1.00	1.00	1.00	1.00
Quartile 2 ^1^	1.19 (0.98, 1.45)	0.97 (0.80, 1.19)	0.82 (0.67, 1.01)	1.03 (0.84, 1.25)	1.01 (0.76, 1.36)
Quartile 3 ^1^	1.19 (0.98, 1.46)	0.80 (0.65, 0.98)	0.84 (0.68, 1.02)	1.01 (0.83, 1.23)	1.12 (0.84, 1.49)
Quartile 4 ^1^	1.29 (1.06, 1.58)	1.24 (1.01, 1.51)	1.11 (0.91, 1.35)	1.40 (1.15, 1.70)	1.91 (1.46, 2.48)
*P*-trend	0.263	0.263	0.560	0.010	<0.001
DHA(C22:6)					
Quartile 1	1.00	1.00	1.00	1.00	1.00
Quartile 2	1.05 (0.86, 1.28)	0.88 (0.72, 1.06)	1.04 (0.85, 1.27)	1.03 (0.85, 1.25)	1.11 (0.86, 1.42)
Quartile 3	0.85 (0.69, 1.04)	0.74 (0.61, 0.91)	0.88 (0.72, 1.08)	0.78 (0.64, 0.95)	0.89 (0.69, 1.16)
Quartile 4	0.80 (0.66, 0.98)	0.53 (0.43, 0.65)	0.80 (0.65, 0.98)	0.60 (0.49, 0.73)	0.54 (0.40, 0.72)
*P*-trend	0.855	<0.001	0.073	<0.001	<0.001
DHA/EPA ratio					
Quartile 1	1.00	1.00	1.00	1.00	1.00
Quartile 2	0.97 (0.79, 1.18)	0.69 (0.56, 0.84)	0.73 (0.60, 0.89)	0.75 (0.62, 0.91)	0.48 (0.37, 0.61)
Quartile 3	0.88 (0.72, 1.08)	0.66 (0.54, 0.81)	0.82 (0.67, 1.01)	0.72 (0.59, 0.87)	0.44 (0.33, 0.57)
Quartile 4	0.72 (0.59, 0.88)	0.70 (0.58, 0.86)	0.79 (0.65, 0.97)	0.64 (0.52, 0.77)	0.51 (0.40, 0.66)
*P*-trend	0.068	0.003	0.185	<0.001	<0.001

Abbreviation: HTG, hypertriglyceridemia; EPA(C20:5), eicosapentaenoic acid (20:5); DHA(C22:6), docosahexaenoic acid(C22:6). ^1^ ORs (95% CI) of MetS for quartile 2, quartile 3, and quartile 4: adjusted for age, gender, education, marital status, income, tea drinking, smoking status, alcohol drinking, multivitamin use, physical activity, daily energy intake, dietary fiber, and SFAs, with quartile 1 as the referent.

**Table 4:** Multivariable-adjusted HRs (95% CIs) for the incidence of metabolic syndrome by quartiles of erythrocyte polyunsaturated fatty acids.

Variables	HRs (95% CI) by Quartiles of *n*-3 PUFA Concentrations				*P*-Trend
	Quartile 1	Quartile 2	Quartile 3	Quartile 4	
EPA(C20:5)					
Median (%)	0.27	0.45	0.73	1.61	
Case/n	171/532	188/537	213/583	194/459	
Crude Model	1.00	1.05 (0.86, 1.30)	1.15 (0.94, 1.40)	1.37 (1.12, 1.68)	0.002
Model 1	1.00	1.05 (0.86, 1.30)	1.13 (0.93, 1.38)	1.28 (1.04, 1.57)	0.015
Model 2	1.00	1.06 (0.86, 1.31)	1.12 (0.91, 1.37)	1.26 (1.02, 1.55)	0.032
DHA(C22:6)					
Median	3.04	4.15	4.89	5.88	
Case/n	178/481	172/482	216/534	200/614	
Crude Model	1.00	0.98 (0.80, 1.21)	1.12 (0.92, 1.37)	0.85 (0.70, 1.04)	0.240
Model 1	1.00	0.99 (0.81, 1.23)	1.12 (0.91, 1.36)	0.87 (0.71, 1.06)	0.294
Model 2	1.00	1.00 (0.81, 1.24)	1.14 (0.93, 1.40)	0.89 (0.72, 1.09)	0.422
DHA/EPA ratio					
Median	2.65	6.72	9.88	13.51	
Case/n	189/452	216/562	209/549	152/548	
Crude Model	1.00	0.89 (0.73, 1.08)	0.86 (0.71, 1.05)	0.63 (0.51, 0.78)	<0.001
Model 1	1.00	0.93 (0.76, 1.13)	0.92 (0.76, 1.13)	0.67 (0.54, 0.83)	<0.001
Model 2	1.00	0.95 (0.78, 1.16)	0.95 (0.78, 1.17)	0.70 (0.56, 0.86)	0.002

Abbreviations: PUFA, polyunsaturated fatty acids; EPA(C20:5), eicosapentaenoic acid (20:5); DHA(C22:6), docosahexaenoic acid(C22:6). Model 1: adjusted for age, gender; Model 2: further adjusted for education, marital status, income, tea drinking, smoking status, alcohol drinking, multivitamin use, physical activity, daily energy intake, dietary fiber, and SFAs.

**Table 5:** Multivariable-adjusted HRs (95% CIs) for the incidence of metabolic syndrome components by quartiles of erythrocyte polyunsaturated fatty acids.

Variables	Abdominal Obesity	HTG	LOW HDL-C	Hypertension	Hyperglycemia
Cases/n	751/1589	736/1973	668/1938	558/1633	924/2519
EPA(C20:5)					
Quartile 1	1.00	1.00	1.00	1.00	1.00
Quartile 2 ^1^	0.99 (0.81, 1.21)	1.02 (0.83, 1.25)	0.97 (0.78, 1.21)	0.81 (0.64, 1.02)	1.00 (0.83, 1.22)
Quartile 3 ^1^	1.06 (0.87, 1.29)	0.94 (0.77, 1.16)	0.98 (0.79, 1.22)	0.84 (0.67, 1.06)	1.19 (0.99, 1.43)
Quartile 4 ^1^	1.10 (0.89, 1.36)	1.10 (0.89, 1.36)	1.03 (0.83, 1.29)	0.88 (0.69, 1.12)	1.24 (1.02, 1.50)
*P*-trend	0.311	0.564	0.780	0.338	0.007
DHA(C22:6)					
Quartile 1	1.00	1.00	1.00	1.00	1.00
Quartile 2	0.98 (0.79, 1.21)	0.99 (0.80, 1.23)	1.02 (0.82, 1.27)	0.82 (0.65, 1.05)	1.09 (0.90, 1.32)
Quartile 3	1.04 (0.85, 1.27)	1.06 (0.86, 1.31)	1.06 (0.86, 1.31)	0.67 (0.53, 0.86)	1.04 (0.86, 1.26)
Quartile 4	0.89 (0.73, 1.10)	0.87 (0.71, 1.08)	0.80 (0.64, 1.00)	0.76 (0.61, 0.96)	1.05 (0.87, 1.26)
*P*-trend	0.392	0.279	0.071	<0.001	0.748
DHA/EPA ratio					
Quartile 1	1.00	1.00	1.00	1.00	1.00
Quartile 2	0.99 (0.80, 1.22)	0.90 (0.73, 1.11)	0.94 (0.76, 1.17)	1.08 (0.85, 1.38)	1.00 (0.83, 1.20)
Quartile 3	0.88 (0.71, 1.09)	0.99 (0.81, 1.22)	1.09 (0.88, 1.36)	0.89 (0.69, 1.14)	0.89 (0.74, 1.07)
Quartile 4	0.93 (0.75, 1.14)	0.80 (0.64, 0.99)	0.90 (0.72, 1.12)	0.96 (0.75, 1.23)	0.79 (0.65, 0.96)
*P*-trend	0.318	0.104	0.637	0.381	0.007

Abbreviations: HTG, hypertriglyceridemia; EPA(C20:5), eicosapentaenoic acid (20:5); DHA(C22:6), docosahexaenoic acid(C22:6). ^1^ ORs (95% CI) of MetS for quartile 2, quartile 3 and quartile 4: adjusted for age, gender, education, marital status, income, tea drinking, smoking status, alcohol drinking, multivitamin use, physical activity, daily energy intake, dietary fiber, and SFAs, with quartile 1 as the referent.

## Data Availability

The raw data supporting the conclusions of this article will be made available by the authors on request due to privacy.

## References

[1] Özmen M., Yersal Ö., Öztürk S., Soysal D., Köseeoğlu M.H. (2014). Prevalence of the metabolic syndrome in rheumatoid arthritis. Eur. J. Rheumatol..

[2] Wormser D., Kaptoge S., Di Angelantonio E., Wood A.M., Pennells L., Thompson A., Sarwar N., Kizer J.R., Lawlor D.A., Nordestgaard B.G. (2011). Separate and combined associations of body-mass index and abdominal adiposity with cardiovascular disease: Collaborative analysis of 58 prospective studies. Lancet.

[3] Saklayen M.G. (2018). The Global Epidemic of the Metabolic Syndrome. Curr. Hypertens. Rep..

[4] Lu J., Wang L., Li M., Xu Y., Jiang Y., Wang W., Li J., Mi S., Zhang M., Li Y. (2017). Metabolic Syndrome Among Adults in China: The 2010 China Noncommunicable Disease Surveillance. J. Clin. Endocrinol. Metab..

[5] Gheldof N., Francey C., Rytz A., Egli L., Delodder F., Bovetto L., Piccardi N., Darimont C. (2022). Effect of Different Nutritional Supplements on Glucose Response of Complete Meals in Two Crossover Studies. Nutrients.

[6] Su K.P., Huang S.Y., Chiu T.H., Huang K.C., Huang C.L., Chang H.C., Pariante C.M. (2008). Omega-3 fatty acids for major depressive disorder during pregnancy: Results from a randomized, double-blind, placebo-controlled trial. J. Clin. Psychiatry.

[7] Lopez-Huertas E. (2012). The effect of EPA and DHA on metabolic syndrome patients: A systematic review of randomised controlled trials. Br. J. Nutr..

[8] Aldhafiri F.K. (2022). Investigating the Role of EPA and DHA on Cellular Oxidative Stress; Profiling Antidiabetic and Antihypertensive Potential. J. Pharm. Bioallied Sci..

[9] Pall M.L., Levine S. (2015). Nrf2, a master regulator of detoxification and also antioxidant, anti-inflammatory and other cytoprotective mechanisms, is raised by health promoting factors. Sheng Li Xue Bao.

[10] Calder P.C. (2017). Omega-3 fatty acids and inflammatory processes: From molecules to man. Biochem. Soc. Trans..

[11] Serhan C.N., Levy B.D. (2018). Resolvins in inflammation: Emergence of the pro-resolving superfamily of mediators. J. Clin. Investig..

[12] Chiang N., Serhan C.N. (2020). Specialized pro-resolving mediator network: An update on production and actions. Essays Biochem..

[13] Dai X.W., Chen Y.M., Zeng F.F., Sun L.L., Chen C.G., Su Y.X. (2016). Association between *n*-3 polyunsaturated fatty acids in erythrocytes and metabolic syndrome in Chinese men and women. Eur. J. Nutr..

[14] Mori T.A., Burke V., Puddey I., Irish A., Cowpland C.A., Beilin L., Dogra G., Watts G.F. (2009). The effects of [omega]3 fatty acids and coenzyme Q10 on blood pressure and heart rate in chronic kidney disease: A randomized controlled trial. J. Hypertens..

[15] Andone S., Farczádi L., Imre S., Bălașa R. (2022). Fatty Acids and Lipid Paradox-Neuroprotective Biomarkers in Ischemic Stroke. Int. J. Mol. Sci..

[16] AbuMweis S., Abu Omran D., Al-Shami I., Jew S. (2021). The ratio of eicosapentaenoic acid to docosahexaenoic acid as a modulator for the cardio-metabolic effects of omega-3 supplements: A meta-regression of randomized clinical trials. Complement. Ther. Med..

[17] Dasilva G., Pazos M., García-Egido E., Pérez-Jiménez J., Torres J.L., Giralt M., Nogués M.R., Medina I. (2016). Lipidomics to analyze the influence of diets with different EPA:DHA ratios in the progression of Metabolic Syndrome using SHROB rats as a model. Food Chem..

[18] Andersen G., Harnack K., Erbersdobler H.F., Somoza V. (2008). Dietary eicosapentaenoic acid and docosahexaenoic acid are more effective than alpha-linolenic acid in improving insulin sensitivity in rats. Ann. Nutr. Metab..

[19] Lluís L., Taltavull N., Muñoz-Cortés M., Sánchez-Martos V., Romeu M., Giralt M., Molinar-Toribio E., Torres J.L., Pérez-Jiménez J., Pazos M. (2013). Protective effect of the omega-3 polyunsaturated fatty acids: Eicosapentaenoic acid/Docosahexaenoic acid 1:1 ratio on cardiovascular disease risk markers in rats. Lipids Health Dis..

[20] Dasilva G., Pazos M., García-Egido E., Gallardo J.M., Rodríguez I., Cela R., Medina I. (2015). Healthy effect of different proportions of marine ω-3 PUFAs EPA and DHA supplementation in Wistar rats: Lipidomic biomarkers of oxidative stress and inflammation. J. Nutr. Biochem..

[21] Wang P., Chen Y.M., He L.P., Chen C.G., Zhang B., Xue W.Q., Su Y.X. (2012). Association of natural intake of dietary plant sterols with carotid intima-media thickness and blood lipids in Chinese adults: A cross-section study. PLoS ONE.

[22] Yang Y.X., Wang G., Pan X. (2002). China Food Composition Table 2002.

[23] Alberti K.G., Eckel R.H., Grundy S.M., Zimmet P.Z., Cleeman J.I., Donato K.A., Fruchart J.C., James W.P., Loria C.M., Smith S.C. (2009). Harmonizing the metabolic syndrome: A joint interim statement of the International Diabetes Federation Task Force on Epidemiology and Prevention; National Heart, Lung, and Blood Institute; American Heart Association; World Heart Federation; International Atherosclerosis Society; and International Association for the Study of Obesity. Circulation.

[24] Ding D., Li Y.H., Xiao M.L., Dong H.L., Lin J.S., Chen G.D., Chen Z.Y., Tang X.Y., Chen Y.M. (2020). Erythrocyte Membrane Polyunsaturated Fatty Acids Are Associated with Incidence of Metabolic Syndrome in Middle-Aged and Elderly People-An 8.8-Year Prospective Study. J. Nutr..

[25] Jang H., Park K. (2020). Omega-3 and omega-6 polyunsaturated fatty acids and metabolic syndrome: A systematic review and meta-analysis. Clin. Nutr..

[26] Woodman R.J., Mori T.A., Burke V., Puddey I.B., Watts G.F., Beilin L.J. (2002). Effects of purified eicosapentaenoic and docosahexaenoic acids on glycemic control, blood pressure, and serum lipids in type 2 diabetic patients with treated hypertension. Am. J. Clin. Nutr..

[27] Mori T.A., Bao D.Q., Burke V., Puddey I.B., Beilin L.J. (1999). Docosahexaenoic acid but not eicosapentaenoic acid lowers ambulatory blood pressure and heart rate in humans. Hypertension.

[28] Yang X., Li X., Hu M., Huang J., Yu S., Zeng H., Mao L. (2024). EPA and DHA differentially improve insulin resistance by reducing adipose tissue inflammation-targeting GPR120/PPARγ pathway. J. Nutr. Biochem..

[29] Kalupahana N.S., Claycombe K., Newman S.J., Stewart T., Siriwardhana N., Matthan N., Lichtenstein A.H., Moustaid-Moussa N. (2010). Eicosapentaenoic acid prevents and reverses insulin resistance in high-fat diet-induced obese mice via modulation of adipose tissue inflammation. J. Nutr..

[30] Yu S., Xie Q., Tan W., Hu M., Xu G., Zhang X., Xie G., Mao L. (2023). Different ratios of DHA/EPA reverses insulin resistance by improving adipocyte dysfunction and lipid disorders in HFD-induced IR mice. Food Funct..

[31] Greupner T., Kutzner L., Nolte F., Strangmann A., Kohrs H., Hahn A., Schebb N.H., Schuchardt J.P. (2018). Effects of a 12-week high-α-linolenic acid intervention on EPA and DHA concentrations in red blood cells and plasma oxylipin pattern in subjects with a low EPA and DHA status. Food Funct..

[32] Flachs P., Horakova O., Brauner P., Rossmeisl M., Pecina P., Franssen-van Hal N., Ruzickova J., Sponarova J., Drahota Z., Vlcek C. (2005). Polyunsaturated fatty acids of marine origin upregulate mitochondrial biogenesis and induce beta-oxidation in white fat. Diabetologia.

[33] Minihane A.M. (2016). Impact of Genotype on EPA and DHA Status and Responsiveness to Increased Intakes. Nutrients.

[34] Bertrand C., Pignalosa A., Wanecq E., Rancoule C., Batut A., Deleruyelle S., Lionetti L., Valet P., Castan-Laurell I. (2013). Effects of dietary eicosapentaenoic acid (EPA) supplementation in high-fat fed mice on lipid metabolism and apelin/APJ system in skeletal muscle. PLoS ONE.

[35] Ghasemi Fard S., Wang F., Sinclair A.J., Elliott G., Turchini G.M. (2019). How does high DHA fish oil affect health? A systematic review of evidence. Crit. Rev. Food Sci. Nutr..

[36] Hashimoto M., Shinozuka K., Gamoh S., Tanabe Y., Hossain M.S., Kwon Y.M., Hata N., Misawa Y., Kunitomo M., Masumura S. (1999). The hypotensive effect of docosahexaenoic acid is associated with the enhanced release of ATP from the caudal artery of aged rats. J. Nutr..

[37] McLennan P., Howe P., Abeywardena M., Muggli R., Raederstorff D., Mano M., Rayner T., Head R. (1996). The cardiovascular protective role of docosahexaenoic acid. Eur. J. Pharmacol..

[38] Pifferi F., Cunnane S.C., Guesnet P. (2020). Evidence of the Role of Omega-3 Polyunsaturated Fatty Acids in Brain Glucose Metabolism. Nutrients.

[39] Mori T.A., Burke V., Puddey I.B., Watts G.F., O’Neal D.N., Best J.D., Beilin L.J. (2000). Purified eicosapentaenoic and docosahexaenoic acids have differential effects on serum lipids and lipoproteins, LDL particle size, glucose, and insulin in mildly hyperlipidemic men. Am. J. Clin. Nutr..

[40] Nakasatomi M., Kim H., Arai T., Hirako S., Shioda S., Iizuka Y., Sakurai K., Matsumoto A. (2018). Fish oil and fenofibrate inhibit pancreatic islet hypertrophy, and improve glucose and lipid metabolic dysfuntions with different ways in diabetic KK mice. Obes. Res. Clin. Pract..

[41] Prostek A., Gajewska M., Bałasińska B. (2016). The influence of eicosapentaenoic acid and docosahexaenoic acid on expression of genes connected with metabolism and secretory functions of ageing 3T3-L1 adipocytes. Prostaglandins Other Lipid Mediat..

[42] Pal A., Metherel A.H., Fiabane L., Buddenbaum N., Bazinet R.P., Shaikh S.R. (2020). Do Eicosapentaenoic Acid and Docosahexaenoic Acid Have the Potential to Compete against Each Other?. Nutrients.

[43] Preston Mason R. (2019). New Insights into Mechanisms of Action for Omega-3 Fatty Acids in Atherothrombotic Cardiovascular Disease. Curr. Atheroscler. Rep..

[44] Liu L., Hu Q., Wu H., Xue Y., Cai L., Fang M., Liu Z., Yao P., Wu Y., Gong Z. (2016). Protective role of n6/n3 PUFA supplementation with varying DHA/EPA ratios against atherosclerosis in mice. J. Nutr. Biochem..

[45] Shang T., Liu L., Zhou J., Zhang M., Hu Q., Fang M., Wu Y., Yao P., Gong Z. (2017). Protective effects of various ratios of DHA/EPA supplementation on high-fat diet-induced liver damage in mice. Lipids Health Dis..

[46] Toth P.P., Chapman M.J., Parhofer K.G., Nelson J.R. (2022). Differentiating EPA from EPA/DHA in cardiovascular risk reduction. Am. Heart J. Plus.

[47] Shimokawa H., Vanhoutte P.M. (1989). Dietary omega 3 fatty acids and endothelium-dependent relaxations in porcine coronary arteries. Am. J. Physiol..

[48] Yin K., Chu Z.M., Beilin L.J. (1991). Blood pressure and vascular reactivity changes in spontaneously hypertensive rats fed fish oil. Br. J. Pharmacol..

[49] Chu Z.M., Yin K., Beilin L.J. (1992). Fish oil feeding selectively attenuates contractile responses to noradrenaline and electrical stimulation in the perfused mesenteric resistance vessels of spontaneously hypertensive rats. Clin. Exp. Pharmacol. Physiol..

[50] McVeigh G.E., Brennan G.M., Cohn J.N., Finkelstein S.M., Hayes R.J., Johnston G.D. (1994). Fish oil improves arterial compliance in non-insulin-dependent diabetes mellitus. Arterioscler. Thromb..

